# Acknowledgment to Reviewers of *Insects* in 2021

**DOI:** 10.3390/insects13020150

**Published:** 2022-01-30

**Authors:** Insects Editorial Office

**Affiliations:** MDPI AG, St. Alban-Anlage 66, 4052 Basel, Switzerland

Rigorous peer-reviews are the basis of high-quality academic publishing. Thanks to the great efforts of our reviewers, *Insects* was able to maintain its standards for the high quality of its published papers. Thanks to the contribution of our reviewers, in 2021, the median time to first decision was 15 days and the median time to publication was 38 days. The editors would like to extend their gratitude and recognition to the following reviewers for their precious time and dedication, regardless of whether the papers they reviewed were finally published:
Aaron GrossJürg EnkerliAaron S. DavidJustin GeorgeAaron T. HaseltonJustin SchmidtAaron TaroneJuvenil Enrique CaresAbdul Hafiz Ab MajidJyrki MuonaAbdulaziz Saad AlqarniK. B. RebijithAbdullahi Ahmed YusufKacem RharrabeAbhishek ChatterjeeKacie AtheyAbraão Almeida SantosKai-Jun LuoAdalécio KovaleskiKaio Cesar Chaboli AleviAdam AlfordKaishu LingAdam James BrunkeKakeru YokoiAdam R. SmithKalle KärhäAdam TofilskiKaloyan IvanovAdekunle AdesanyaKamil HolýAdler R. DillmanKang HeAdnan A. IsmaielKaoru MaetoAdolfo Cordero-RiveraKaren KapheimAdrian BrücknerKaren MenuzAdrian ŁukowskiKaren SalazarAdrian SmolisKarin KirchgatterAdriana Elena MarvaldiKarina WieczorekAdrien FónagyKarl RobinsonAdrienne BrundageKarl RoederAgata KaczmarekKarla AddessoAgostina GiacobinoKarol Barragán FomsecaAi KitazumiKarol GiejdaszAkanksha SinghKarolina FurtakAlain PasquetKasie RaymannAlan DorinKatarzyna BartosikAlan G. GoodmanKatarzyna KmiećAlazne Díez-FernándezKatarzyna Otulak-KoziełAlbane VilarinoKate M. BarnesAlberto ArabKathleen R. WalkerAlberto BiscontinKathleen WestbyAlberto SattaKathrin BrownAlbino Antonio BentoKathrin F. Stanger-HallAldo Dal PràKatrin KellnerAlejandro Del Pozo-ValdiviaKatsuya IchinoseAlejandro ResciaKaty ReedAleš DolnýKaushalya G. AmarasekareAlessandra Della TorreKazuhito AsanoAlessandro CiniKazuki MiuraAlessandro GrapputoKazumu KuramitsuAlessia GiannettoKazunori MatsuoAlessio SavianeKe DongAlex TorsonKeiko Kadono-OkudaAlexander B. RuchinKelly S. JohnsonAlexander BerestetskiyKen FunayamaAlexander KirejtshukKen SasakiAlexander KnyshovKeng-Lou James HungAlexander KuprinKen-Ichi HaranoAlexander MikheyevKenneth A. HayesAlexander SutinKent DaaneAlexandra BeliavskaiaKent S. ShelbyAlexey A. PolilovKentaro ArikawaAlexey I. MakuninKeping ChenAlexey MoseykoKerry WilkinsonAlexey V. ShavrinKeshava MysoreAlfred Edward SzmidtKevin A. WilliamsAlfred M. HandlerKevin B. TemeyerAlfredo Jimenez-PerezKevin R. HinsonAlica KocisovaKevin W. WannerAlice LacinyKikombo NgoyAlida JanmaatKim A. HoelmerAlina AvanesyanKim A. MedleyAlison GrayKim JensenAlison Luce-FedrowKishan SambarajuAlison R. GerkenKi-Woong NamAllan HruskaKiyoshi SaigusaAllan Smith-PardoKlaus HartfelderAllison HansenKohei OguchiAlma SolisKoichi GokaAlon SilberbushKoji TojoAlvaro FuentealbaKoji TsuchidaÁlvaro Rodríguez GonzálezKok-Boon NeohAlvin M. SimmonsKonrad FiedlerAmalia AnthousiKonstantin NadeinAmalio Santacruz VarelaKornelia SkibińskaAman PaulKostas KougioumoutzisAmanda D. RoeKoutaro Ould MaenoAmanda SorensenKrijn PaaijmansAmeya D. GondhalekarKrishnendu MukherjeeAmir AyaliKristine L. WerlingAmit RoyKristopher L. GilesAna CrnkovicKrzysztof MilerAna Luísa Coderniz PicançoKrzysztof SzpilaAna Luisa García-PérezKsenia S. OnufrievaAna Piedra-BuenaKwang-Zin LeeAnamika SharmaKyeong-Yeoll Lee‪Anand SinghKyle M. BenowitzAnastasia LevitinKyriaki VarikouAnat Levi-ZadaKyudong HanAnatoly SavelievKyung-Jin MinAnders AakKyu-Sik ChangAndre Luis Da Costa Da SilvaLajos RozsaAndré Morgado EstevesLan ZhangAndre WilkeLance DurdenAndrea BattistiLara MaistrelloAndrea BecchimanziLarissa Almeida MartinsAndrea BirkeLaszlo TretterAndrea SciarrettaLaura BortolottiAndrea SquartiniLaura DepaloAndrea TóthováLaura FergusonAndreas KrugerLaura GrasaAndrei AlyokhinLaure Desutter-GrandcolasAndrei LegalovLauren WeidnerAndrei SourakovLaurence A. MoundAndrew CherrillLaurent VuatazAndrew D. SweetLav SharmaAndrew G. S. CuthbertsonLawrence HribarAndrew GutierrezLe KangAndrew J. RosendaleLeandro Rodrigues SantiagoAndrew JohnsonLech KarpińskiAndrew K. DavisLee CohnstaedtAndrew PolaszekLeila GasmiAndrew WhittingtonLekhnath KafleAndrey Alexandrovich LegalovLeon G. HigleyAndrzej GrzywaczLeonardo AncillottoAndrzej OleksaLeonardo M. TurchenAneta A. PtaszyńskaLeslie C. LewisAneta StracheckaLeslie S. Saul-GershenzAngel GuerreroLewis BartlettAngel Roberto BarchukLewis Vibul HunAngela MeccarielloLi ChenAngela PrendinLiang JiangAngela RoggeroLidia LimontaAngela SantilioLi-Hsin WuÁngeles AdánLincoln SmithAngelika HilbeckLinda MasonAngelina Wójcik-FatlaLinda ThomsonAngelique VetillardLindsey C. PerkinAngelita AcebesLing TianAngelo CanaleLing-Hsiu LiaoAniello FrancescoLionel FrangeulAnita GiglioLisa TimmonsAnita LiškaLise RoparsAnn HorsburghLivia ZapponiAnn StockerLiviu-Adrian CotfasAnna GajdaLiwei WuAnna HeitmannLizette L. KoekemoerAnna HimlerLong YangAnna HonkanenLorella GiovannelliAnna PapachLoren J. Rivera-VegaAnna Rodolfa MalacridaLorenzo PrendiniAnna SimonettoLorenzo ToninaAnna StöcklLoris GalliAnnabelle FirlejLouis E. N. JackaiAnnalisa De BoniLubomir KovacAnneke A. VeenstraLuc BelzuncesAnthia MatsakidouLuc LegalAntje Dietz-PfeilstetterLuc P. BelzuncesAntoine FeldenLuc SweversAntoinette P. MalanLuca FacchinelliAnton StabentheinerLuca FinettiAntonella Di PalmaLuca Pietro CasacciAntonia ChroniLuca RuiuAntonia Garrido FrenichLucia Adriana Escudero ColomarAntonio BelcariLucia SantorufoAntonio CarapelliLuciana GalettoAntonio Eduardo Miller CrottiLuciano BossoAntonio FelicioliLucy AlfordAntonio MasettiLuigi RiganoAntonio NanettiLuis Ángel Rubio San MillanAntonio OrtizLuis Carlos MartínezAntonio Pietro GaronnaLuis EspinoAntonio RicarteLuis F. AristizábalAntonio ZuritaLuis Fernando ChavesAntonios AugustinosLuís Filipe Prazeres BonifácioAntonios MichaelakisLuis Gustavo Amorim PessoaAntonios TsagkarakisLuis J. ChuecaApostolos KapranasLuis Miguel De PablosArathi SeshadriLuis Miguel Torres-VilaArezki IzriLuís NetoArgiro KalaitzakiLuis R. PertierraAriane DorŁukasz KajtochAriel ChipmanLuke JacobusArinder K. AroraLuke M. JacobusAris IliasLuke R. TembrockArjen E. Van ’t HofLumi ViljakainenArkadiusz UrbańskiLynn M. RiddifordArne BeermannLyric BartholomayArne WeinholdMadhavi L. KakumanuAron D. KatzMagdalena BrodaArthur G. AppelMaite VillanuevaArthur ShapiroMaki N. InoueArun RajamohanMakio TakedaAry HoffmanMakoto TokudaAsad SultanMalayka Samantha PicchiAsgar EbadollahiMałgorzata DmitryjukAshish RawsonMałgorzata ŁochyńskaAshley E. LarsenMałgorzata MajewskaAshley LeachManabu KamimuraAstrid EbenManfred HartbauerAtashi SharmaMani ShresthaAthanasios ZervasManisha KolakshyapatiAthanassios D. VelentzasManoj NayakAthanassios K. GiatropoulosManon AugusteAtsuo YoshidoManuela PlutinoAttila GereManuela RamalhoAtul Kumar PandeyManuela ReboraAugusto LoniManuela Rodrigues BrancoAulo ManinoMan-Yeon ChoiAurora MontaliMarc BenedictAurore Avarguès-WeberMarc S. HalfonAwawing Anjwengwo AndongmaMarcela BucekovaAxel KalliesMarcela Carina AudisioAxel SchwerkMarcela LareschiBabar HassanMarcela MoréBahar Md HabibullahMarcela RodrigueroBalint KacsohMarcella Windmuller-CampioneBalog AdalbertMarcelo Andrés Umsza-GuezBaohua XuMarcelo Mendes RabeloBaosheng ZengMárcia S. CouriBao-Zhen HuaMarcio Martinello SanchesBaptiste MartinetMarco FerranteBarbara ContiMarco MeneguzBarbara FeldmeyerMarco PezziBarbara Nieradko-IwanickaMarco PietropaoliBarbara Van AschMarco PorporatoBarrett A. KleinMarco SalveminiBartlomiej TroczkaMarcos Antonio Matiello FadiniBartosz KierończykMarcos Roca-CusachsBartosz PłachnoMarcus Alvarenga SoaresBeata Borowiak-SobkowiakMarek JindraBeata GabryśMarek MichalskiBeata GrzywaczMargareth Lara CapurroBeata HoreckaMargarita OrlovaBeatrice N. DinghaMari H. OgiharaBeatriz Sabater-MuñozMaria BoukouvalaBeatriz Sevilla-MoránMaria Cristina FotiBenjamin B. NormarkMaria Fernanda AchinellyBenjamin CullMaría García-GuzmánBenjamin FuerstenauMaría Gloria Sáenz-RomoBenjamin LinardMaria KonstantopoulouBenjamin PelissieMaria LazarinaBenoit GeslinMaria Luisa DindoBenoit TalbotMaría MorazaBernard P. DufourMaria NilssonBernard RoitbergMaria Otilia CarvalhoBernard StaniecMaría Pérez-MarcosBernhard HausdorfMaría Shantal Rodríguez-FloresBhadriraju H. SubramanyamMaria Teresa RebeloBinu AntonyMaria Vittoria ManciniBob MinckleyMaría-Isabel ArnaldosBonk Kyu ByunMariana S. CastroBoyd A. MoriMariangela BonizzoniBožena BarićMariano Higes PascualBradley J. SinclairMaribel R. PortillaBrandyce St LaurentMaribet GamboaBrenna E. TraverMarie C. PizzornoBrett M. SeymoureMarie PizzornoBrian D. EitzerMarie Vestergaard HenriksenBrian FisherMarina AscunceBrian G. RectorMarina J. Orlova-BienkowskajaBrian V. BrownMarina VilenicaBrian ViaMarino QuarantaBrittany CapbellMario Alejandro MarínBrittany F. PetersonMário BoieiroBrittany S. BarkerMario D’AmicoBrook O. SwansonMárió GajdácsBruce E. HibbardMario ManzoBruno BagnoliMario Segio PalmaBruno BelliniMario VersaciBruno MassaMariusz KanturskiBruno R. S. FigueiredoMariusz TrytekBruno RossaroMark BelkBruno Rossitto De MarchiMark C. HarrisonBryan GiordanoMark E. MankowskiByju Nambidiyattil GovindanMark MankowskiByron N. Van NestMark Q. BenedictCai WangMark SchwarzlaenderCaio A. Leal-DutraMarko MutanenÇakmak İbrahimMarko ProusCam DonlyMarkus FriedrichCameron E. WebbMarkus W. EitleCamilla R. SharkeyMarta Skowron VolponiCarina ZittraMartijn J. T. N. TimmermansCarl LowenbergerMartín AlujaCarlo DusoMartin FikáčekCarlo PolidoriMartin GiurfaCarlos Alfredo Lopes De CarvalhoMartin H. VilletCarlos BarcelóMartin HolmstrupCarlos CaceresMartin KonvičkaCarlos Lopez-VaamondeMartin KulmaCarlos P. SilvaMartin ŠigutCarlos Pascacio-VillafánMartin StreinzerCarmelo Peter BonsignoreMartyna K. ZwoinskaCarmen CallejasMary L. CorneliusCarmen QueroMaryia MishynaCarolina Gomez-DiazMarylène PoiriéCaroline ZanchiMasahito T. KimuraCasper VanderwoudeMasako KatsukiCassandra KohMasami MasumotoCatarina Prado E. CastroMasanao SatoCatherine LittleMassimiliano VirgilioCaven Mguvane MnisiMassimo CristofaroCerian WebbMatan ShelomiCésar Chávez Galarza JúlioMateus CamposCesar Rodriguez-SaonaMathilde GendrinChanglu WangMatija GogalaChangqi LiuMatteo DaineseChao ChenMatteo MarcantonioChaoyang ZhaoMatthew A. CollinCharalampos ProestosMatthew D. TragerCharity G. OwingsMatthew DoremusCharles AppersonMatthew F. RouhierCharles Barry KnisleyMatthew GruwellCharles BartlettMatthew J. BallingerCharles BurksMatthew S. JonesCharles J. StuhlMatthew S. LehnertCharles M. MacVeanMatthew W. TurnbullCharles MarshMatthieu GuichardCharles R. VossbrinckMatti KoivulaCharley Christian StaatsMattias ForshageCharlotte LécureuilMaurilia Maria MontiChaz HyseniMaurizio BrivioCheng SunMaurizio MuzziChengshu WangMauro FoisChenyang CaiMauro Manuel Martinez PachecoCheyenne TaitMauro MarrelliChi K. NguyenMaxence GérardChiara FerraciniMaxwell J. ScottChieka MinakuchiMayra Cadorin VidalChihiro HimuroMazhar HussainChin-Yuan HsuMd Habibullah BaharChin-Cheng Scotty YangMeg AllenChin-Tong OngMegha KalsiChris BosioMeghan O. Grady MilbrathChris DickmanMelina CamposChristian CambillauMelissa Anne JohnsonChristian FigueroaMelissa D. JordanChristian StaufferMelody A. KeenaChristian Tellgren-RothMeng MaoChristian WegenerMerko VagaChristie S. HerdMette-Cecilie NielsenChristina L. MogrenMichael A. NashChristina MüllerMichael BrownbridgeChristina PilzMichael C. SingerChristine A. NalepaMichael CaterinoChristine DieckhoffMichael DomingueChristine StruckMichael GarveyChristoph HoffmannMichael HrncirChristophe BressacMichael I. SitvarinChristophe DaugeronMichael J. BrewerChristophe DominikMichael J. GoblirschChristophe HanoMichael J. PitcairnChristophe LucasMichael J. RaupachChristopher A. VarnonMichael J. SamwaysChristopher AdamsMichael Jay GrodowitzChristopher CarltonMichael Joseph StoutChristopher H. DietrichMichael KearneyChristopher J. GedenMichael KošťálChristopher M. RangerMichael KristensenChristopher R. StephensMichael L. MayChristopher RangerMichael PeckChristopher W. WeldonMichael RaupachChristos G. AthanassiouMichael RedingChristos I. RumbosMichael RiehleChukwunonso O. NzeluMichael RoeChuleui JungMichael RustChun-Sen MaMichael S. CrossleyChunsheng HouMichael ScharfChunxia WangMichael SchmittClaire DumenilMichael SergeevClaudia A. SzentiksMichael SparksClaudia GanserMichael SternClaudia GarridoMichael W. BelitzClaudia KeilMichael WilsonClaudinéia Pereira CostaMichael WinterbournCláudio José Von ZubenMichal KnappClaus P. W. ZebitzMichał KrzyżowskiCleber GalvãoMichal MotykaClélia OlivaMichal SegoliClement F. KentMichal ZurovecClément SchneiderMichel FaucheuxCongfen GaoMichel RenouCorey BrelsfoardMichel SartoriCorey L. CampbellMichelangelo La SpinaCorrado BattistiMichele QuintanoCostanza JuckerMichele RicuperoCraig R. MacadamMicheline K. StrandCrisalejandra Rivera-PérezMichelle FountainCristina CastaneMichelle J. SolenskyCsaba SzinetárMidori TudaCynthia D. Scott-DupreeMiguel Angel MirandaCynthia LordMiguel López-FerberCyril PiouMiha HumarDagmara ŻyłaMika MurataDaisuke KageyamaMike ToewsDakshina R. SealMilan PernekDale A. HalbritterMilana S. MitrovicDalibor TiteraMilosz MazurDalton De Souza AmorimMing BaiDamien CharabidzeMing-Cheng WuDan BeanMing-Long YuanDana L. PriceMingluen JengDana MentMinmin CaiDan-Dan ZhangMirella Lo PintoDaniel DoucetMiroslav BartákDaniel JanzenMitzy F. PorrasDaniel K. YoungMohamed AlburakiDaniel KlineMohamed G. NasserDaniel ParedesMohamed Z. M. SalemDaniel R. HowardMohammed BakkaliDaniel R. SuiterMohsen M. RamadanDaniel WinklerMokhtar Abdulsattar ArifDaniela MiricescuMonica RosenbluethDaniela PilarskaMonika StenglDaniela RandoMuhammad HaseebDaniele AlberoniMuhammad QasimDaniele Da ReMuhammad Z. AhmedDaniele PorrettaMurray B. IsmanDaniele SommaggioMyron ZaluckiDanilo Oliveira CarvalhoNadezhda OstroverkhovaDanival José De SouzaNadia MuscoDanon Clemes CardosoNaïla EvenDaochao JinNana Y. AnkrahDara StocktonNancy EndersbyDaria BajerleinNancy McintyreDarija LemicNancy OstiguyDariusz IwanNaoya OsawaDariusz PiesikNarkis S. MoralesDasiel ObregonNatália RaschmanováDave TonkynNatalia V. LebedevaDavid A. IngberNatalie Hempel de IbarraDavid AngeliniNatalie WestDavid B. BuchwalterNatalya A. KuznetsovaDavid BaracchiNathan ButterworthDavid BeresfordNathan L. HaanDavid C. LeesNatraj KrishnanDavid F. CookNaves PedroDavid FurthNawaf Al-MaharikDavid HaymerNeeraj SoniDavid HunterNeil AudsleyDavid I. Shapiro-IlanNelson SimõesDavid Keith YeatesNena PavlidiDavid L. HuNestor O. Nazario-YepizDavid M. DonnellNetta DorchinDavid M. LehmannNicholas C. ManoukisDavid MerrittNicholas JohnsonDavid MoriñaNicholas KomarDavid Mota-SanchezNicholas M. TeetsDavid NagyNicholas RomanoDavid PearsonNick LarsonDavid PlotkinNicola A. IrvinDavid RileyNicola BodinoDavid Robert HallNicola SimcockDavid W. HagstrumNicolas MontagnéDavid W. InouyeNicolas NègreDavid Ward RoubikNicole F. QuinnDavid WariNicole R. SextonDavide BertelliNicoletta FaraoneDavide RassatiNiels PiotDeborah Ann WallerNigel RaineDeborah RotoliNigel StrawDeby L. CassillNikita KlugeDedong LiNikolaos I. KatisDeepak B. Thimiri Govinda RajNikos E. PapanikolaouDenis J. WrightNils HeinDenise SelivonNils Th. GrabowskiDennis PaulsNina DeisigDennis PaulsonNina JenkinsDennis WelkerNoah RoseDiana M. LeemonNorbert BeckerDiana Perez-StaplesNoriaki IijimaDiego Fernando SeguraNorma Robledo QuintosDiego GallegoNorman L. BeattyDiego SaukaNuray BaserDimitrios N. AvtzisOksana SkaldinaDina Orozco-DavilaOlavi KurinaDing YangOldřich NedvědDingze MangOleksii SkorokhodDionysios Ch. PerdikisOlga M. C. C. AmeixaDmitry E. ShcherbakovOlga PawełczykDmitry KolomenskiyOlga YaroslavtsevaDmitry TelnovOliver KellerDoekele G. StavengaOlivier SparaganoDolors BoschOmaththage P. PereraDomenico CarminatiOmaththage PereraDonald B. ThomasÖmer ErtürkDonald C. WeberOndřej BalvínDonald James McLeanOscar AlomarDonald R. StrongOvidiu Alin PopoviciDonald WeberOwen LonsdaleDonatella BattagliaPablo LiedoDonato MarianoPablo MontoyaDong Soon KimPaloma Mas-PeinadoDong ZhangPanagiotis A. EliopoulosDonn JohnsonPanagiotis G. MilonasDora HenriquesPanagiotis J. SkourasDora MarinovaPaola SangiorgioDorcas OrengoPaolo A. PedataDori Edson NavaPaolo FantiDoris Lagos-KutzPaolo GabrieliDouglas ZeppeliniPaolo GrattonDouglass H. MorsePaolo PastorinoDrion G. BouciasParide DioliDushyant MishraPascal QuernerE. Jorge TizadoPatrice BouchardEddie UeckermannPatricia G. TillmanEddy DowlePatrícia ThyssenEdmundo SousaPatrick A. GuerraEduard VivesPatrick Anthony GuerraEduardo Fuentes-ContrerasPatrick De ClercqEduardo Gabriel VirlaPatrick FerreeEdward DevesonPatrick J. MoranEdward F. ConnorPatrick KellyEdwin R. BurgessPatrick MoranEileen A. HebetsPatrizia FalabellaEileen KnorrPaul AirsEirik SøvikPaul AyayeeEk Del ValPaul B. BakerEkaterina V. GrizanovaPaul BrakefieldEl Desouky AmmarPaul EadyEleftherios AlissandrakisPaul EggletonElena CostiPaul GrundyElena DrosopoulouPaul J. DysonElena GonellaPaul-André CalatayudElena L. ZverevaPaulo A. V. BorgesElena López-GallegoPavel DobešElena RhodesPavel SaskaElena RomeraPaweł GlibowskiElia StahlPedro Da SilvaElijah TalamasPedro GuiraoElisa GarzoPedro YamamotoElisabetta RossiPei LiangElisângela De Souza LoureiroPei-Shi YenElison Fabricio Bezerra LimaPenelope GreensladeElizabeth BeersPeng LüElizabeth HenryPengcheng LiuElsa BonnaféPepijn W. KooijElsa Borges SilvaPerttu SeppaEmanuele MazzoniPetar J. KljajićEmil Engelund ThybringPeter ChandlerEmiliano MoriPeter H. AdlerEmily J. BairdPeter JamesEmily R. Burdfield-SteelPeter KlepsatelEmma DesplandPeter MayoEmma GillinghamPeter McGheeEmma WeeksPeter RidlandEmmanuel Viana PontualPeter SchausbergerEn-Cheng YangPeter WitzgallEnrico De LilloPetr BoguschEnrico RuzzierPetr KmentEnrique Baquero MartinPetr PyszkoEran LevinPetros T. DamosEric CaragataPhil SuterEric ContiPhilip C. WheaterEric DarrouzetPhilip KohlmeierÉric GuilbertPierre GiovenazzoEric Harold CliftonPierre OuvrardEric JangPietro BrandmayrEric PalevskyPing WangEric S. TvedtePiotr CeryngierEric W. ChambersPiotr SzwedaEric Wellington RiddickPolychronis RempoulakisEricsson David Coy-BarreraPompeo SumaErik T. FrankPrabodh SatyalErika PlettnerPrasad ParadkarErika TuckerPriyadarshini ChakrabartiErin D. TreanorePriyanka MittapellyErin L. McCulloughQiao WangErin ScullyQilian QinErnst WimmerQingjun WuErrol HassanQiong YangEsmaeil AmiriQisheng SongEsmat HegaziQuentin SzaboEsteban Rodriguez-LeyvaQuentin ThommenEstève BoutaudRachael HornerEsther LanteroRachel A. ArangoEthel M. VillalobosRachel M. MohrEugene RyabovRadek AulickyEugenia E. MontielRadomir JaskułaEugênio Eduardo De OliveiraRafael JordanaEustachio TarascoRafael RodriguezEvan P. KelemenRafał BernardEvelyne HougardyRafał GosikEverlyne WosulaRaffaele GriffoEvgeniy MakarchenkoRaheleh RavanfarEvgeniy S. BalakirevRahman SarthokEwa TomaszewskaRaimondas MozūraitisEzequiel GonzalezRajinder S. MannEzio PeriRalem Gary ChiangF. Ortiz-SánchezRalf EinspanierFabiana EspositoRalf NauenFabienne RibeyreRalitsa BalkanskaFabio CianferoniRamzi MansourFabio Laurindo Da SilvaRaquel Martín-HernándezFabrício PereiraRasa BernotienėFabrizio BallestrinoRassim Rassim KhelifaFaith KuehnRaul Alberto LaumannFan SongRebeca RosengausFarley William Souza SilvaRebecca Trout FryxellFatemeh GanjisaffarRegiane Cristina Oliveira De Freitas BuenoFátima AmaroRegiane Maria Tironi De MenezesFederico CappaReina TongFederico LessioReinhold SiedeFei YangRenata BažokFelicity MuthRené SforzaFeng CuiRicardo BessinFeng WangRicardo MachadoFeng ZhangRicardo Pérez-De La FuenteFengming YanRicardo Siqueira Da SilvaFerdinand Nanfack-MinkeuRichard FellFerdinando Di MartinoRichard KarbanFerenc LakatosRichard MarchantFerenc TóthRichard OdemerFernando Ariel GentaRichard S. PeiglerFernando CanovasRichard S. ZackFernando Teruhiko HataRichard W. MankinFilipe MadeiraRichard WilkersonFilippe Elias De Freitas SoaresRid MargitFilippo FrizziRika OzawaFilitsa KaramaounaRina HamajimaFinbarr HorganRita MarulloFinn KjellbergRita RebolloFlavio Henrique-SilvaRobert A. JohnsonFlávio Roberto Mello GarciaRobert BodeFletcher YoungRobert D. Mitchell IIIFlore MasRobert F. SmithFlorian MenzelRobert HarrisonFranca RossiRobert J. HigginsFrances S. SivakoffRobert J. OrpetFrancesc Gomez-MarcoRobert KruegerFrancesca BeriniRobert L. MinckleyFrancesca LaudaniRobert MeagherFrancesca ManciantiRobert Michael PyleFrancesca MariniRobert NofemelaFrancesco NardiRobert S. VargovitshFrancesco NugnesRoberto BarreraFrancesco PaoliRoberto CaldaraFrancesco ParisiRoberto KellerFrancesco PorcelliRoberto LigroneFrancesco SeverinRoberto MannuFrancesco SpinelliRoberto RomaniFrancinaldo Soares SilvaRobin KundrataFrancis Morais Franco NunesRodrigo KrugnerFrancisca RuanoRoger A. LaineFrancisca SegersRoger BlackmanFrancisco BeitiaRoghaiyeh KarimzadehFrancisco Díaz-FleischerRollie J. ClemFrancisco FerreiraRoma DurakFrancisco Sánchez-BayoRomain LibbrechtFranco MutinelliRomain NattierFrançois LorenzettiRoman BorovecFrançois PayreRoman BucherFrank ByrneRoman ModlingerFrank-Thorsten KrellRoman V. YakovlevFred EllerRoman Viktorovich YakovlevFred R. MusserRomano DallaiFrédéric FrancisRoméo BelliniFrédéric G. BrunetRomoli OttaviaFrédéric LabbéRosa GálvezFrédéric Marion-PollRosângela BarbosaFrédérique A. O. HilliouRoseli La CorteFredric V. VenclRosemary Susan LeesFrolov AndreyRoshan ManandharFuji JianRosilda M. MussuryFumio HayashiRosilda Mara MussuryFumio YokohariRoy G. Van DriescheG. Millar JocelynRuan VeldtmanGabor L. LöveiRubi Nelsi Meza LázaroGábor LöveiRudolf H. ScheffrahnGabriel HughesRuggero PetacchiGabriela Castaño-MenesesRui Cardoso-PereiraGabriela Pérez-LachaudRui GuoGabriele GrecoRui-De XueGabriele RondoniRunzhi ZhangGabriella Lo VerdeRupinder KaurGaëlle Le GoffRustem A. IlyasovGaetana MazzeoRyan KueselGaetano SiscaroRyo NakaoGail AndersonRyota HosomiGaku TokudaRyszard SzadziewskiGalen DivelyRyuta UrakiGao HuSaad ArifGarance Di PasqualeSabrina BertinGareth J. LycettSalima Machkour-M’RabeGarrett LeagueSalvatore BellaGary B. DunphySalvatore GuarinoGary BrewerSamuel BrownGary M. BarkerSandhya MishraGema NietoSándor KeszthelyiGen KanekoSándor KoczorGene E. RobinsonSandra AllanGene Sheng TungSandra RojasGeoff MorseSandra VacasGeoff OxfordSandrine A. LacourGeoffrey LynnSang Soo LeeGeoffrey M. AttardoSang-Bin LeeGeorge D. BroufasSantolo FrancatiGeorge G. KennedySantosh RevadiGeorge MbataSan-Yuan MaGeorge T. KoliopoulosSaptarshi GhoshGeraldo CarvalhoSara ArgandaGérard DemangeatSara L. BushmannGerardo R. CamiloSarah C. WoodGertha WilhelmSarah Page LawsonGéza RipkaSascha BuchholzGiacinto Salvatore GerminaraSatoshi HiroyoshiGianfranco AnforaSauro SimoniGianluca TettamantiSaveer AhmedGilberto José De MoraesSchöller MatthiasGil-Hah KimScott FitzgeraldGiliane De Souza TrindadeSean BlamiresGillian EastwoodSebastian GisderGillian GileSebastian PitaGina AngelellaSebastian SalataGiobbe ForniSengottayan Senthil-NathanGiorgia GiordaniSenta NiedereggerGiorgia SollaiSeong Hwan ParkGiorgio BinelliSergey ReznikGiovanna PonteSergey Ya ReznikGiovanni BurgioSergey Yurievich StorozhenkoGiovanni CiliaSergi MaicasGiovanni MariniSergio MagallanesGiovanni SogariSergio MiglioreGiselher GrabenwegerSergio Pérez-GuerreroGiulia GiuntiSérgio RodriguesGiulia MagogaSeth N. RedmondGiuseppe Eros Massimino CocuzzaSeverin HattGiuseppe Massimino CocuzzaShah FahadGiuseppe MazzaShaku NairGiuseppino Sabbatini-PeverieriShaohui WuGleb N. ArtemovShao-Ji HuGlenn E. StudebakerShaojin WangGongyin YeShao-Kang HuangGonzalo A. AvilaShaoli WangGordon PortSharavari KulkarniGraham A. McCullochShaukat AliGregg HendersonSheina KofflerGregorio VonoShengzhang DongGregory A. DahlemShimat JosephGregory J. DaglishShin-Ichi AkimotoGregory LoebShlomo NavarroGregory ZolnerowichShruti YadavGrit KunertShugeng WuGuang YangShuji ItakuraGuanhong WangShüné OliverGuaraci Duran CordeiroShunhua GuiGuilherme DiasSidnei Alves De AraujoGunnar BrehmSigfrid IngrischGunnar IsacssonSilvana PiersantiGuohua HuangSilvia CasiniGuo-Hua ZhongSilvia Teresa MoraglioGuoqing WeiSimon Luke ElliotGustavo TaretSimon McKirdyGuy JosensSimon TierneyGwen PearsonSimon W. BaxterH. John B. BirksSimona SagonaH. Michael G. LattorffSimone BellucoHabibu MugerwaSimone FattoriniHaeyong KweonSimone RosaHaidong DingSiva Kumar ChamarthiHajnalka SzentgyörgyiSkarlatos G. DedosHana SehadováSławomir MitrusHanano YamadaSofia G. SeabraHannes SchulerSofie MeeusHans Peter RavnSónia DuarteHans-Peter FuehrerSônia N. BáoHany M. R. Abdel-LAtifSoo Hyun ParkHaobo JiangSoo Jean ParkHaralabos TsolakisSoraia I. FalcãoHariet HinzSpecht AlexandreHarpal SinghSpyros SfenthourakisHarvey LemelinSrećko ĆurčićHeather Christine BellSreepriya PramodHeather L. LeachSriyanka LahiriHeather McAuslaneStanislav TrdanHéctor A. VargasStará JitkaHéctor González HernándezStefania BalzanHee Il LeeStefania LaudoniaHeidi ConnahsStefano BediniHeikki HokkanenStefano CivolaniHélcio Gil-SantanaStefano VaninHeleen P. KrugerStefanos AndreadisHelene LeBlancStefanos LeontopoulosHelmut KÄFERStephen A. MarshallHenk SiepelStephen CookHerbert Júnior DiasStephen J. FletcherHerbert VenthurStephen L. CameronHeverton DutraSteve WhyardHideharu NumataSteven C. CookHidehiro WatanabeSteven J. RiceHideto HoshinaSteven P. BradburyHidetoshi TeramotoSteven PresleyHiroe YasuiSteven T. PeperHiroko UdakaSteven TrewickHiroshi AbéSteven Van BelleghemHiroyuki YoshitomiSteven Van TimmerenHitoshi TsujimotoSubhas HajeriHong PangSudip GaireHongbo JiangSukovata LidiaHong-Gang TianSulaiman Sadi IbrahimHong-Lei WangSurya SahaHonglin FengSusan BrownHongzhang ZhouSusan C. JonesHu LiSusan GrunerHu WanSusan T. HarbisonHuabing WangSuzanne E. BlattHuahua SunSven BradlerHubert AmreinSwapna Priya RajarapuHubert CharlesSylvia AntonHugh A. SmithSylwia StączekHugo A. BenítezSzymon MatuszewskiHugo Alejandro BenitezTae-Sung KwonHui WangTaiyo YoshiokaHui YuTakahiro KikawadaHume B. DouglasTakao ItiokaHuoqing ZhengTakashi KiuchiHyojoong KimTakeshi FujiiHyun-Woo ParkTakeshi MiuraIago Sanmartín VillarTakuya UeharaIain PatersonTalbot TrotterIan KaplanTamar KeasarIan ScottTamara BotoIda Chapaval PimentelTamara Nunes Lima-CamaraIgnazio FLORISTan KenIgor MalenovskýTânia Mesquita NobreIgor UspenskyTanja McKayIgor V. ShamshevTanya RussellI-Hsin SungTapan BhattacharyyaIke OlivottoTara D. GariepyIkju ParkTatjana KramaIlaria Negri Ted E. CottrellIlias KioulosTeng LiIndrė LipatovaTeodor RusuIndrikis KramsTerence BradshawInga ReichTerence L. BradshawInka LusebrinkTeresa BonacciInmaculada Garrido-JuradoTeresa Palomeque MessíaInon ScharfTeresa VasconcelosIoana GrozeaTerezia HorvathovaIrene MuñozTetsuo GotohIrene PicciniTetsuya BandoIsaac EderyTheeraphap ChareonviriyaphapIsaac L. EsquivelTheo ZeegersIsabel Martinez-SañudoTherese PolandIsabela Galarda VarassinThibaud MonninIsabelle DietrichThibaut DelsinneIsmail DokerThierry BourgoinIstvan AndoThies H. BüscherItamar GlazerThinandavha Caswell MunyaiIvan HiltpoldThomas A. O'Shea-WhellerIvan MilosavljevićThomas AssmannIvana Tlak GajgerThomas BawinIves HaifigThomas C. WebsterJ. Christopher BerghThomas DuboisJ. Joe HullThomas E. ReaganJ. Spencer JohnstonThomas G. SheltonJaap De RoodeThomas HesselbergJacek FrancikowskiThomas J. HenryJacek KamczycThomas KroeberJacek SzwedoThomas M. PerringJacek TwardowskiThomas N. VassilakosJacinto Benhadi-MarínThomas SpranghersJacob CorcoranThomas Van De KampJacob WengerThomas W. PhillipsJacob WickhamThomas WassmerJader De OliveiraTian RenmaoJai Singh PatelTiantao ZhangJakob WegenerTim George ShreeveJames B. BurrittTimo DomischJames E. AlexanderTimothy A. EbertJames F. WalgenbachTimothy JuddJames M. W. RyallsTimothy LysykJames MainoTimothy MousseauJames Montoya-LermaTimothy R. FallonJames R. OttTimothy W. MouralJames S. WatersTing LiJames SucklingTing-Wu ChuangJames TauberTobias EnglJames ZahniserTobias LiljaJan BuellesbachTodd M. GilliganJan E. ConnTolulope A. AgunbiadeJan KleckaTom HillJan LubawyTom WeihmannJan ŠevčíkTomáš DitrichJan ŠobotníkTomáš LacknerJan VotypkaTomislav MašekJana C. LeeTommaso CampaniJana CollatzTong-Xian LiuJane CostaToomas TammaruJanez PrešernTorben StemmeJaret DanielsTorbjørn EkremJarkko RouttuToru TogawaJaroslav HolušaToshiharu MitaJarosław BrykToshiharu TanakaJaroslaw KrzywinskiTravis W. RuschJaroslaw SklodowskiTrevor WilliamsJason H. ByrdTrisna TungadiJason L. MotternTse-Yu ChenJason L. RobinsonTsuneyuki TatsukeJason M. SchmidtUlrich IrmlerJay A. RosenheimUlrich Rainer ErnstJean Luc GattiUmberto BernardoJean Luc GattolliatVaclav StejskalJean Marcel DeguenonValentina G. KuznetsovaJean TurgeonValentina MastrantonioJean-François PicimbonValeria TrivelloneJean-Luc BoevéValéria Wanderley-TeixeiraJean-Michel GibertValerie CaronJean-Pierre LumaretValerio SbordoniJean-Yves RasplusVance G. NielsenJedeliza FerraterVanessa S. DiasJeff DavisVanessa Simões DiasJeff GarnasVassiliki I. EvangelouJeffrey D. WellsVassilis DourisJeffrey LittlefieldVenera I. TyukmaevaJeffrey OliverVera A. KrischikJeffrey TomberlinVera S. SorokinaJennifer A. BrissonVernard LewisJennifer K. PetersonVeronica AndreoJennifer KrauelVeronica Calles-TorrezJennifer M. JandtVeronica M. SinotteJennifer MorrowVictor Benno Meyer-RochowJens AmendtVictor Manuel Sanchez-CorderoJeongjoon AhnVíctor Rogelio Castrejón-GómezJeremy B. JewellVictoria SorokerJeremy C. AndersenVid BakovicJeremy V. CampViktor HartungJernej PolajnarViktor V. BrygadyrenkoJerome A. HogsetteVincent CorbelJerome MurienneVincent DietemannJeronimo AlencarVincent ForayJerry HogsetteVincenzo CavalieriJerzy A. LisVincenzo NataleJerzy RomanowskiVinton ThompsonJerzy WildeVladimir A. LukhtanovJesper G. SørensenVladimir GokhmanJessica D. HohensteinVladimir HulaJessica DittmerVladimír KošťálJessica WareVolker FramenauJesús AvillaVonnie ShieldsJesús Gómez-ZuritaW. Robert ShawJesús Jiménez-RuizWaleska VeraJevrosima StevanovicWalter SandtnerJia HuangWannes DermauwJian DuanWanxue LiuJiang ZhongWanzhi CaiJiangfeng WangWaqa WakilJianhong LiWardatou BoukariJianing WuWarren P. PorterJianya SuWayne Brian HunterJia-Wei TayWee L. YeeJiayong ZhangWei XuJiayue YanWei YuJibin ZhangWeihua MaJie ShenWeining ChengJilian LiWen LiuJill LancasterWen-Po ChuangJinshun ZhongWen-Wu ZhouJinsong ZhuWes WatsonJiří NermutWiebke SickelJiri SkuhrovecWiktoria SzydłoJoachim R. De MirandaWill BouchardJoanna N. IzdebskaWilliam Benjamin WalkerJoanna WerszkoWilliam D. BrownJoanne Y. YewWilliam EtgesJoao Aristeu Da RosaWilliam N. SetzerJoaquin Gomis-CebollaWilliam ReidJoaquin L. Reyes LópezWilliam TangJoaquín Ortega-EscobarWojciech GrodzkiJocelyn G. MillarWolf Christian LuzJochen ZeilWolfgang BlenauJoh R. HenschelWolfram GrafJohanna E. ElsensohnWolfram MeyJohanne BrunetWon Il ChoiJohannes Human GiliomeeWoojin KimJohannes KoehbachWouter DekoninckJohannes L. M. SteidleWuchun TuJohannes StraussXia LeeJohn A. ByersXianhui WangJohn AcornXiao ChenJohn Allen ShueyXiaobo WuJohn C. MorseXiaofei TuJohn EwerXiaofeng WuJohn GoolsbyXiao-Feng XueJohn J. ObryckiXiaolei HuangJohn Kevin MoultonXiaoling TongJohn M. PleasantsXiaoping WangJohn McCreadieXiaotian TangJohn O. Stireman IIIXiao-Yong ChenJohn Paul MutebiXifeng WangJohn RubersonXin ZhouJohn ShorterXingeng WangJohn SkartveitXing-Jia ShenJohn T. MargaritopoulosXingtan ZhangJohn TrumbleXingzhong LiuJohn WeissXinzhi NiJohnnie Van Den BergXuankun LiJoji OtakiXueyan LiJolanta Bro$\tilde{z}$ekYadong HuangJonas KupplerYael D. LubinJonathan A. CammackYan KongJonathan DarbroYan ZhangJorddy Neves CruzYasuhiko MatsumotoJordi RiudavetsYeon Jae BaeJorge A. GuimarãesYidong WuJorge Ari NoriegaYing YanJorge CancinoYingbo MaoJorge Paredes MonteroYizhou ChenJörn TheuerkaufYoko InuiJosé A. P. MarcelinoYoko TakasuJosé Carlos FrancoYong Hun JoJosé Dijair AntoninoYongjun ZhangJosé Éduardo SerraõYongqi ShaoJosé Enrique González-ZamoraYooichi KAINOHJosé GaliánYoonseong ParkJosé López ColladoYoosook LeeJose Luis Anaya-LópezYoshiaki HashimotoJosé Maurício Simões BentoYoshinobu HayashiJosé Max Barbosa De Oliveira-JúniorYoshinori MutoJose NegronYou LiJose PietriYouming HouJose RamirezYuanzhen LiuJosé Ricardo Lima PintoYudai NishideJose TudelaYueping HeJoseph KaserYue-Wen ChenJoshua GibsonYu-Liang YangJouni SorvariYu-Lingzi ZhouJovana BozicYun MaJowita DrohojowskaYunke WuJoyce M. SakamotoYuri N. ShkrylJozef J. M. Van Der SteenYuriy IlinskiyJozsef FailYu-Shin NaiJózsef KissYutaka BannoJozsef RohacsYuval Gottlieb-DrorJozsef VutsYu-Xuan YeJuan MorroneYuyu WangJuang-Horng ChongYves RoisinJuanita Rodriguez ArrietaZachary ShafferJudith M. StahlZaira Valentina ZizzariJukka SalmelaZdenek Faltynek FricJukka SuhonenŽeljko TomanovićJulia D. FineZemek RostislavJulia EbelingZeyang ZhouJulia FerrarZhaozhi LuJulia GrasslZhen ZouJulia M. MalyshZhen-Ying WangJulian HillyerZhiguo LiJulie CarcaudZhi-Wei LiuJulien PetillonZhongshi ZhouJulio Rodriguez-AndresZilá Luz Paulino SimõesJulio Vicente Figueroa-MillánZoltán ElekJun TabataZoltan ImreiJun XuZoltán S. VargaJune-Sun YoonZoran StanimirovicJunhuan XuZsolt BalintJuraj MedoZuzana Kronekova


